# Experimental data on convective drying of potato samples with different thickness

**DOI:** 10.1016/j.dib.2018.04.065

**Published:** 2018-04-24

**Authors:** I. Boutelba, S. Zid, P. Glouannec, A. Magueresse, S. Youcef-ali

**Affiliations:** a Laboratoire de Génie Climatique (LGCC), Université Frères Mentouri Constantine 1, Ain Elbey, 25000 Constantine, Algérie; b Institut de Recherche Dupuy de Lôme-SEPT, Université Européenne de Bretagne, B.P.92116, 56321 Lorient Cedex, France

## Abstract

A laboratory setup is developed to examine the characteristics of convective drying of a moist potato sample. The thickness effect (5, 10 and 15 mm) of product on heat and mass transfer during potato drying was presented. Potato samples were dried at temperature of 51 °C and air velocity of 1 m s^−1^. Structural Change (length, width and thickness) of the samples were measured during drying. The heat distribution at different locations, sides surfaces and heart of the samples, was acquired at a time interval of 30 min for the test period. According to drying rate, potato drying is predominantly in the falling rate period. The moisture diffusivity data determined from experimental convective drying kinetics. The method based on the analytical solution of a fickian diffusive model has been developed to both evaluate the sample thickness effects on moisture diffusivity. Moreover, this dataset is made public in order to be used by other researchers studying the performances analysis of the drying systems.

**Specifications Table**

**Table:** 

Subject area	Food engineering
More specific subject area	Drying
Type of data	Table, image, figure
How data was acquired	To measure the mass loss, the potato sample is suspended to a digital balance of a precision of ± 0.01 g. However, to measure the temperature evolution two thermocouples of K type with a precision of ± 10^−^^2^ °C are used to measure the temperature in the center and in the left side, a pyrometer Optris CS with a precision of ± 0.1 °C is used to measure the temperature of the right side. The structural changes (length, width and thickness) were measured at different locations and drying times using a digital Calipers [1]. The effective moisture diffusivity coefficient was estimated by combined the drying kinetics with the second Fick's law [2].
Data format	Raw, analyzed
Experimental factors	The potato samples used in experiments were cut into rectangular slices having the different thickness (5, 10 and 15 mm), the samples will undergo neither bleaching nor another treatment.
Experimental features	Tests were carried out on the potato sample drying. The data acquired were compared to determine the thickness effect on the drying time and the moisture diffusivity in the sample.
Data source location	Laboratoire du Génie Climatique Constantine, Université Frères Mentouri Constantine 1, Constantine, Algérie; Institut de Recherche Dupuy de Lôme, Université Bretagne Sud, Lorient, France
Data accessibility	With this article

**Value of the data**

• The data can be used to investigate the effect of thickness on the drying time and the moisture diffusion.
• The dataset of the temperature and drying rate can be used to determine the different drying periods.
• The dataset of the dimensions potato samples can be used to determine the shrinkage and density.
• The drying rate data it's necessary to determine the evaporated mass flux.
• The dataset can be used by other researchers studying the performances analysis of the drying systems.

## 1 Data

**Fig. 1:**
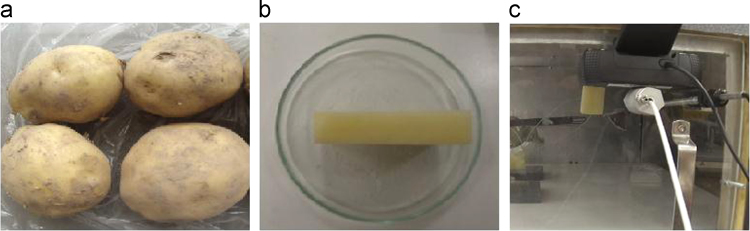
Photographs show the preparation product steps: (a) raw potato; (b) potato sample; (c) instrumentation sample (sample in dryer).

**Fig. 2:**
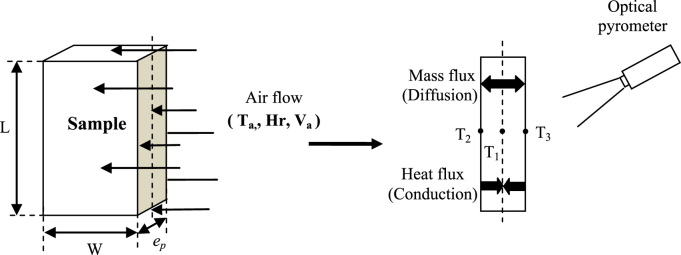
Sample details.

**Fig. 3:**
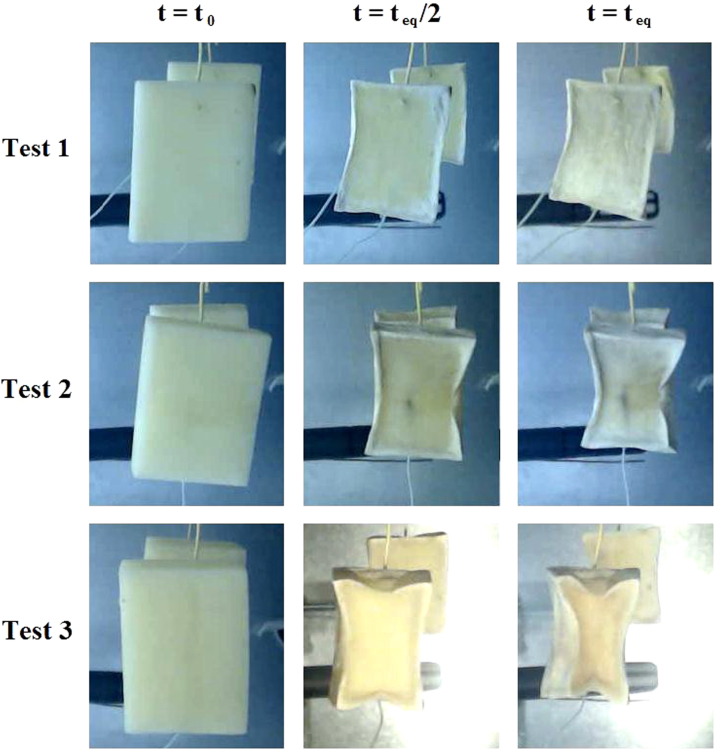
Photographs potato samples of different tests at the beginning, half and the end of drying.

**Fig. 4:**
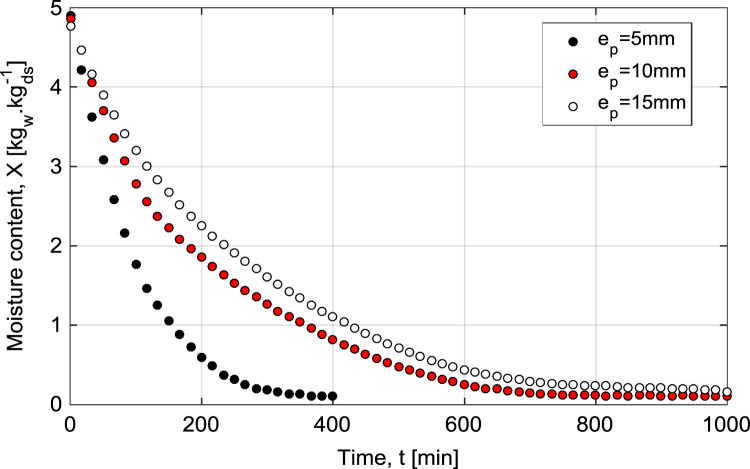
Evolution of mean moisture contents as a function of time at different thickness.

**Fig. 5:**
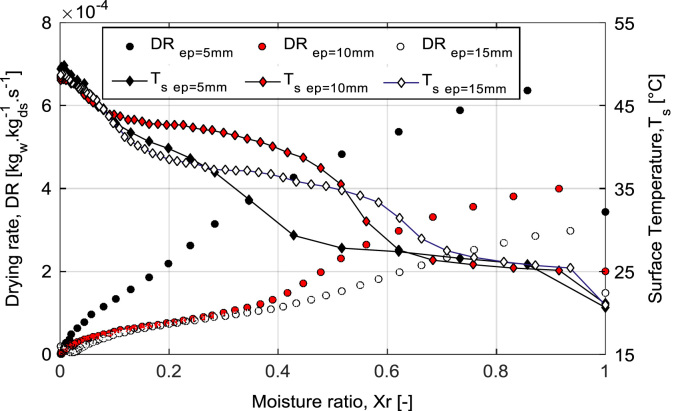
Kinetic drying of potato samples at different thickness.

**Fig. 6:**
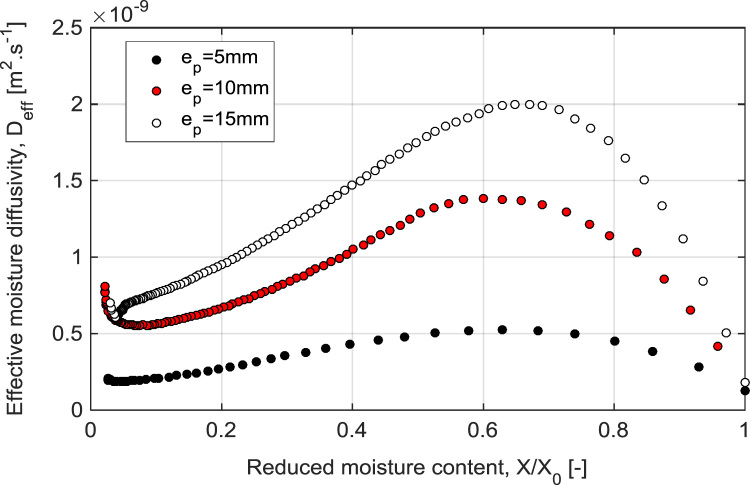
Effective moisture content diffusivity coefficient at different thickness.

The Mean experimental conditions and samples initial dimensions was gathered in Table 1. The Mean measured dimensions of different experiment are reported in Table 2. Table 3, Table 4, Table 5 summarizes the experimental data of different tests. This data contains valuable information of temperature air and samples thus that loss mass product. The Fig. 1, Fig. 2, Fig. 3 shown the instrumentation procedure and the surface evolution of potato samples from different tests. In Fig. 4, the characteristic drying curves are depicted for different thickness. These data were used to evaluate moisture diffusivity as described in Section 2.4. Whereas, the drying rate and surface temperature of samples are plotted in Fig. 5. After an initial short warming-up period the drying rate reached a maximum value and then it followed by three falling rate period in all experiments. Estimated water diffusivities are plotted in Fig. 6. The thickness sample influence water mobility in the potato, where the moisture diffusivity data increase with the thickness [3]. Also, as cited in the literature [4], [5], the *D*_*eff*_ values reached a maximum between *X*/*X_0_* = 0.6–0.7 and then decreased as drying progresses.

**Table 1:** Mean experimental conditions and samples initial dimensions.

**Exp. number**	**Air temperature*****T***_***a***_**(°C)**	**Air velocity*****V***_***a***_**(m/s)**	**Air humidity*****Hr*****(%)**	**Thickness*****e***_***p***_**(mm)**	**Width *W*(mm)**	**Length *L* (mm)**	**Initial mass *m****_**0**_***(g)**
**1**	51	1	7.7	5.16	30.15	39.95	6.80
**2**	51	1	7.6	10.15	30.29	40.18	14.47
**3**	51	1	8	15.15	30.18	40.12	20.06

**Table 2:** Mean measured dimensions of different experiment.

**Time (min)**	**Mean dimension Test 1**			**Mean dimension Test 2**			**Mean dimension Test 3**		
	**Thickness*****e***_***p***_**(mm)**	**Width *W*(mm)**	**Length *L*(mm)**	**Thickness*****e***_***p***_**(mm)**	**Width *W*(mm)**	**Length *L* (mm)**	**Thickness*****e***_***p***_**(mm)**	**Width *W*(mm)**	**Length *L*(mm)**
**0**	5.16	30.15	39.95	10.15	30.29	40.18	15.15	30.18	40.12
**30**	4.15	28.38	37.78	9.27	28.04	39.04	13.97	29.78	39.42
**60**	3.35	26.78	36.81	8.52	27.81	37.82	12.91	29.05	38.48
**90**	2.80	25.59	35.11	7.84	26.98	36.92	12.02	28.14	37.54
**120**	2.45	24.79	33.86	7.20	26.42	36.31	11.24	27.48	36.48
**180**	1.71	24.12	32.62	6.02	25.37	35.22	9.80	26.52	35.42
**240**	1.42	23.72	31.85	4.94	24.33	34.53	8.62	25.62	34.81
**300**	1.36	23.41	31.32	4.04	23.75	33.92	7.31	24.81	34.15
**360**	1.33	23.36	31.24	3.48	23.31	33.02	6.84	24.28	33.66
**420**	–	–	–	3.28	23.04	32.49	6.18	23.61	33.18
**540**	–	–	–	3.21	22.82	32.19	5.13	22.74	32.41
**660**	–	–	–	3.17	22.73	32.02	4.53	22.18	31.68
**840**	–	–	–	3.16	22.67	31.92	4.32	22.04	31.23
**1020**	–	–	–	–	–	–	4.28	21.96	31.15

**Table 3:** Experimental data showing test 1.

**Time (min)**	Average air velocity = 1 m s^−1^					
	Average air humidity = 7.7%					
	***T**_**a**_***(°C)**	***T***_**2**_**(°C)**	***T***_**3**_**(°C)**	***T***_**4**_**(°C)**	***T***_**1**_**(°C)**	**Mass (g)**
**0**	50.81	20.69	20.4	20.54	20.30	6.80
**30**	51.03	26.61	25.8	26.21	25.62	5.45
**60**	51.25	27.56	27.8	27.68	27.02	4.35
**90**	51.20	31.58	34.5	33.04	33.03	3.43
**120**	51.33	37.35	39.4	38.37	38.29	2.78
**150**	51.44	39.84	41.4	40.62	40.53	2.36
**180**	51.28	41.45	43.5	42.47	42.6	2.02
**210**	51.53	43.85	46.1	44.98	45.49	1.75
**240**	51.21	47.09	48.4	47.75	48.02	1.54
**270**	51.27	48.08	48.9	48.49	48.87	1.41
**300**	51.51	48.66	49.3	48.98	49.31	1.35
**330**	51.45	49.11	49.6	49.36	49.80	1.29
**360**	51.37	49.59	49.9	49.74	50.03	1.28
**390**	51.30	49.18	49.5	49.34	49.7	1.27

**Table 4:** Experimental data showing test 2.

**Time (min)**	Average air velocity = 1 m s^−1^					
	Average air humidity = 7.6%					
	***T**_**a**_***(°C)**	***T***_**1**_**(°C)**	***T***_**2**_**(°C)**	***T***_**3**_**(°C)**	***T***_**4**_**(°C)**	**Mass (g)**
**0**	51.43	21.08	21.14	20.7	20.92	14.47
**30**	51.29	25.04	25.39	24.6	24.99	12.69
**60**	51.28	25.62	26.08	25.6	25.84	11.14
**90**	51.03	27.89	28.38	29.1	28.74	9.73
**120**	51.28	36.42	35.96	36	35.98	8.67
**150**	51.31	39.12	38.72	38.8	38.76	7.95
**180**	51.35	40.28	39.89	39.9	39.89	7.36
**210**	51.21	41.33	40.85	40.6	40.72	6.85
**240**	51.36	42.14	41.50	41.3	41.40	6.38
**270**	51.15	42.97	42.09	41.7	41.89	5.95
**300**	51.18	43.58	42.33	41.9	42.11	5.57
**330**	51.21	44.06	42.54	42.1	42.32	5.22
**360**	51.28	44.52	42.74	43.1	42.92	4.87
**390**	51.43	44.95	42.98	43.3	43.15	4.56
**420**	51.33	45.45	43.37	44	43.67	4.27
**450**	51.09	45.85	43.71	44.6	44.15	4.01
**480**	50.97	46.11	43.94	45	44.47	3.78
**510**	50.91	46.43	44.60	46.2	45.40	3.58
**540**	50.89	47.20	45.62	46.9	46.26	3.37
**570**	50.81	47.70	46.61	47.5	47.05	3.20
**600**	50.87	47.96	47.47	47.5	47.49	3.08
**630**	51.03	48.02	47.68	47.9	47.79	2.97
**660**	51.04	48.22	48.09	48.3	48.19	2.86
**690**	50.79	48.38	48.27	48	48.13	2.82
**720**	50.82	48.41	48.20	48.2	48.20	2.77
**750**	50.53	48.41	48.35	48	48.17	2.75
**780**	50.45	48.49	48.42	47.9	48.16	2.74
**810**	50.55	48.58	48.54	48.1	48.32	2.73
**840**	50.44	48.84	48.74	48.1	48.42	2.73
**870**	50.65	48.60	48.65	47.9	48.27	2.72
**900**	50.49	48.01	48.07	47.8	47.93	2.72
**930**	50.54	48.32	48.39	47.9	48.14	2.71
**960**	50.49	48.49	48.56	48	48.28	2.71
**990**	50.52	48.42	48.53	48.3	48.41	2.70

**Table 5:** Experimental data showing test 3.

**Time (min)**	Average air velocity = 1 m s^−1^					
	Average air humidity = 8%					
	***T**_**a**_***(°C)**	***T***_**1**_**(°C)**	***T***_**2**_**(°C)**	***T***_**3**_**(°C)**	***T***_**4**_**(°C)**	**Mass (g)**
**0**	51.51	20.66	20.99	20.5	20.75	20.06
**30**	51.28	25.11	25.65	24.8	25.22	18.12
**60**	51.47	25.85	26.49	25.5	25.99	16.47
**90**	51.49	27.56	28.00	27.2	27.60	14.94
**120**	51.12	32.52	32.07	31.7	31.88	13.75
**150**	51.21	34.63	34.15	33.5	33.83	12.71
**180**	51.29	35.80	35.11	34.6	34.85	11.80
**210**	51.29	36.83	35.76	35.4	35.58	11.01
**240**	50.95	37.96	36.58	36.6	36.59	10.28
**270**	51.10	38.52	36.85	37.2	37.02	9.62
**300**	51.22	39.21	37.16	37.7	37.43	9.03
**330**	51.15	39.53	37.30	37.9	37.60	8.47
**360**	50.84	40.45	37.86	38.7	38.28	7.93
**390**	50.74	40.94	38.21	39.2	38.70	7.43
**420**	50.85	41.38	38.55	39.7	39.12	6.99
**450**	50.72	41.84	39.25	40.4	39.82	6.55
**480**	50.59	42.36	40.07	41.4	40.73	6.15
**510**	50.58	43.33	41.13	42.5	41.82	5.82
**540**	50.65	44.36	42.50	44.1	43.30	5.48
**570**	50.52	45.36	43.98	45.4	44.69	5.21
**600**	50.60	45.87	45	46.4	45.7	4.97
**630**	50.71	46.73	46.05	47.2	46.62	4.80
**660**	50.59	47.06	46.56	47.4	46.98	4.62
**690**	50.70	47.24	46.98	47.6	47.29	4.50
**720**	50.49	47.42	47.24	47.9	47.57	4.42
**750**	50.68	47.41	47.29	48.2	47.74	4.34
**780**	50.89	47.66	47.54	48	47.77	4.30
**810**	50.72	47.62	47.6	48.2	47.9	4.28
**840**	50.66	47.67	47.64	48.2	47.92	4.22
**870**	50.74	47.90	47.93	48.3	48.11	4.18
**900**	50.65	47.89	47.90	48.6	48.25	4.16
**930**	50.61	47.92	47.99	48.7	48.34	4.15
**960**	50.77	48.03	48.07	48.8	48.44	4.12
**990**	50.88	48.40	48.44	48.4	48.42	4.06
**1020**	51.00	48.37	48.49	48.7	48.59	3.95
**1050**	51.38	48.61	48.7	48.8	48.75	3.82

*T_a_* = Air temperature in dryer.

*T*_1_ = Middle temperature sample.

*T*_2_ = Surface temperature of left side.

*T*_3_ = Surface temperature of right side (pyrometer).

*T*_4_ = Average surface temperature = $\left(\frac{T_{2}+T_{3}}{2}\right)$.

## 2 Experimental design, materials and methods

### 2.1 Product

Fresh potato of the same variety "Ondine" (Fig. 1(a)) are used for the experiments, in order to maximize the reproducibility of measurements. For each experiment, the parallelepiped sample (Fig. 1(b)) have been extracted from potato heart (dimensions in Table 1), which allow as to have an homogeneous initial moisture contents and temperatures, knowing that samples will undergo neither bleaching.

### 2.2 Methods

A laboratory scale, horizontal flow dryer was used for the tests. Before each experiment, the temperature and velocity of air in chamber are stabilized for 2 h, in order to achieve steady state conditions. The potato samples are instrumented in a way that all surfaces are exposed to air (see Fig. 1(c)). The experimental conditions are summarized in Table 1. The dry product mass is measured by putting dried product in a vacuum oven at a temperature of 70 °C during 48 h. Weighs were made every 8 h to confirm mass equilibrium.

### 2.3 Moisture content and drying rate

The moisture content for each experiment showing in Fig. 4 was calculated from the dry matter and the weighings reported in Table 3, Table 4, Table 5 using Eq. (1)

(1) $$X=\frac{m_{t}-m_{d}}{m_{d}}\;$$

The drying rates (DR) of potato samples during the drying experiments showing in Fig. 5 were calculated by using the following equation [6], [7]:

(2) $$DR=\frac{d\left(m_{t+dt}-m_{t}\right)}{dt}\;$$

where *m*_*t*_ is the dry matter at any time *t* (kg), *m*_*d*_ is the dry matter (kg), *m*_*t+dt*_ is the dry matter at time *t+dt* (kg)

To obtain the drying rate data, it's necessary to go through a numerical processing on the derivative of the mass in order to overcome the error. At first, the time step is lengthened during the derivation [8]. After lengthening the time step to 500 s, filtering is performed by a MATLAB program using the function "smooth (y)".

### 2.4 Effective diffusivity coefficient

The effective diffusivity coefficient was estimated by second Fik's law, according to the Eq. (3) proposed by Crank [9]

(3) $$Xr=\frac{8}{\pi^{2}}\sum_{n=0}^{\infty }\frac{1}{{\left(2n+1\right)}^{2}}\;exp\;\left(-{\left(2n-1\right)}^{2}\frac{\pi^{2}D_{eff}t}{4\;{e_{p}}^{2}}\right)$$

where *D*_*eff*_ is the effective moisture diffusivity (m^2^ s^−^^1^), *e*_*p*_ is the half of thickness, and *t* is drying time (s).

The moisture ratio (*Xr*) of potato samples during the drying experiments were calculated by using the following equation:

(4) $$Xr=\frac{X_{t}-X_{eq}}{X_{0}-X_{eq}}$$

where *X*_*t*_ is the moisture content at a specific time (kg_w_ kg _ds_^−^^1^), *X*_*0*_ is the initial moisture content (kg_w_ kg _ds_^−1^), *X*_*eq*_ is the equilibrium moisture content (kg_w_ kg _ds_^−1^)

Based on assumptions of constant moisture diffusivity and thickness on of short time periods. Knowing that on these periods, the effect of temperature and shrinkage on mass transfer is neglected. The solution of Eq. (3) is thus obtained by a step-by-step method using an optimization procedure under Matlab software.
